# Structural Perspectives of Insulin Receptor Isoform-Selective Insulin Analogs

**DOI:** 10.3389/fendo.2017.00167

**Published:** 2017-07-27

**Authors:** Jiří Jiráček, Lenka Žáková

**Affiliations:** ^1^Institute of Organic Chemistry and Biochemistry, Czech Academy of Sciences, Prague, Czechia

**Keywords:** insulin receptor isoform, IR-A, IR-B, CT-peptide, exon 11, insulin analog, binding affinity

## Abstract

A significant drawback of the exogenous administration of insulin to diabetics is the non-physiological profile of insulin action resulting in the insufficient suppression of hepatic glucose production, which is the main contributing factor to diabetic hyperglycemia under fasting conditions and the basis of the challenge to restore a more physiological glucose profile in diabetes. The insulin receptor (IR) exists in two alternatively spliced variants, IR-A and IR-B, with different tissue distribution. While peripheral tissues contain different proportions of both isoforms, hepatic cells almost exclusively contain IR-B. In this respect, IR-B-selective insulin analogs would be of great interest for their potential to restore more natural metabolic homeostasis in diabetes. Recent advances in the structural biology of insulin and IR have provided new clues for understanding the interaction of both proteins. This article discusses and offers some structural perspectives for the design of specific insulin analogs with a preferential binding to IR-B.

Insulin has been used in the treatment of diabetes for almost a century, due to the seminal achievements in 1922 by the teams of Banting, Best, McLeod and Collip in Toronto, ON, Canada. In 2014, there were an estimated 422 million adult diabetics globally, compared to 108 million in 1980. The global prevalence (age-standardized) of diabetes has nearly doubled since 1980, rising from 4.7 to 8.5% of the adult population. Insulin is the essential life-saver of type 1 diabetics (7–10% of all diabetics). However, it is also necessary for almost 20% of type 2 diabetics (World Health Organization). Besides human insulin, specifically modified insulins, insulin derivatives or analogs, are given to patients, offering them more convenience and safety. Rapid-acting analogs with an onset of action several times faster than natural human insulin can be administered shortly before a meal. On the contrary, long-lasting analogs act for several hours and are usually taken overnight, while intraperitoneally (i.p.) administered human insulin is cleared from the circulation in less than 2 h. It is important to mention, that both rapid-acting and slow-lasting analogs owe their properties primarily to their structural features or additives in their formulations, which determine their release to the circulation as active monomers rather than to different kinetics of interaction of their monomers with the insulin receptors (IRs) (1–4).

If administered orally, insulin as a protein hormone is rapidly degraded in the digestive track. Hence, despite intensive efforts to develop strategies for oral insulin administration [e.g., in 2013, Novo Nordisk A/S announced an investment of up to 3.7 billion dollars for the development of an insulin pill by 2020, www.reuters.com (Novo Nordisk decided to scrap the insulin pill program due to commercial hurdles in 2016)] and the approval for two inhalable insulin derivatives [Exubera (poor sales led Pfizer to withdraw Exubera in 2007) and Afrezza], the subcutaneous injection of insulin either by insulin pen or by insulin pump is the most frequent mode of insulin administration (1). Upon subcutaneous administration, a solution of insulin is injected under the skin where it often precipitates or is bound to serum proteins, and insulin is then slowly released primarily to the periphery of the body in the proximity of adipose and muscle tissues. This is in clear contrast to the physiological action of insulin, when the hormone is secreted from the pancreas directly into the portal vein which carries the insulin to the liver. The main action of insulin in the liver is to inhibit gluconeogenesis and glycogenolysis (5). The uptake of glucose into hepatocytes is not mediated by the insulin-regulated GLUT4 transporter, which is the main glucose transporter in adipose and muscle tissues, but by the GLUT2 transporter, which is not insulin-regulated (6, 7). The first action of insulin is thus to block endogenous glucose synthesis when the sugar is available from food. It is estimated that the liver clears about half of the naturally secreted insulin. The second half is then available for its action in peripheral tissues including the brain. On the other hand, the injection of exogenous insulin subcutaneously (s.c.), and consequently its primary action in the periphery, results in insufficient suppression of hepatic gluconeogenesis, which is the main contributing factor to diabetic hyperglycemia under fasting conditions, while impaired glucose uptake to muscle and adipose tissues is important in postprandial hypoglycemia. Therefore, the development of hepatoselective insulin analogs would be of great importance for their potential to restore more natural metabolic homeostasis (5). In this respect, a short-acting insulin analog LY2605541 (or peglispro) with a native amino acid sequence ProB28-LysB29 changed for LysB28-ProB29 swap and an extra 20-kDa polyethylene glycol moiety was promising. The analog reduced renal filtration and prolonged half-life. The increase in molecular size of the analog appeared to alter its tissue distribution in favor of liver versus peripheral tissues (8). However, in 2015, Eli Lilly and Company made the decision to halt the peglispro development program, because the phase III trials reported elevated alanine transaminases and increased fat in the liver (9).

Insulin elicits its functions through binding to the IR, which exists in two isoforms, IR-A and IR-B, resulting from the alternative splicing of the IR gene. The only difference between the isoforms is the 12-amino acid insert (IR-B plus and IR-A minus 12 amino acids) at the C-terminus of the extracellular α-subunit, called the αCT peptide. This differential sequence, which represents only a small fragment of IR (1,380 amino acids in total), has a relatively subtle impact on ligand binding and intracellular signaling, but which may nevertheless have important physiological consequences. Both isoforms have different tissue distribution with the longer IR-B being the largely predominant form in adult humans in hepatocytes (more than 90%), skeletal muscle and subcutaneous fat (both about 70% IR-B) (10), while the shorter IR-A is almost exclusively expressed in other tissues (e.g., brain, lymphatic tissues, or embryo) [Ref. (11) and the references herein]. Thus, insulin analogs with a preferential binding to IR-B could have increased or preferential hepatic bioactivity *in vivo* and could be significant in the treatment of diabetes, with a more physiological profile of action.

Insulin and similar insulin-like growth factors 1 and 2 (IGF-1 and IGF-2), together with their receptors, IR-A, IR-B and the receptor for IGF-1 (IGF-1R), form a complex system which plays a major role in the regulation of basal metabolism, growth, development, healing, lifespan (12–15). In addition, it has a role in the development of cancer (16), diabetes, and other diseases (17). Due to the dimeric character of the receptors (α_2_β_2_ structure), IR-A, IR-B, and IGF-1R can form what are known as hybrid receptors, consisting of one αβ subunit pair from one receptor and the second αβ pair from another receptor. Hybrid receptors have been detected in all tissues and cell lines that express both receptor types (18). Only the IR-A/IR-B hybrid is effectively activated by insulin. The other hybrids (IR-A/IGF-1R and IR-B/IGF-1R) respond 20 times more effectively to IGF-1 than to insulin (19, 20). This phenomenon can have important implications in insulin resistance and the development of type 2 diabetes. Moreover, the insulin-IGF system is further modulated by a family of six IGF-binding proteins (21) and a structurally distinct receptor for IGF-2 (IGF-2R) (22). The typical binding affinities for hormones and their receptors from our laboratory (23–25) are summarized in Table 1, which shows that the main difference between IR-A and IR-B is in their affinities for IGF-1 and IGF-2 but not for insulin. It is not excluded that rather minor differences in insulin *K*_d_ values for IR-A and IR-B shown in Table 1 could be due to some differences in methodologies of cell-based binding assays for IR-A (in membranes of floating IM-9 lymphocytes) and for IR-B (in membranes of adhesive 3T3 fibroblasts), and that absolute binding affinities of insulin for IR-A and IR-B receptors are the same or very similar. It is important to mention that binding affinities of hormones for their receptors measured in different laboratories may slightly differ as well. Once again, this can be due to different methodologies of binding assays (e.g., membrane bound versus soluble receptors, ^125^I- versus Europium-labeled tracers etc.). For example, Denley et al. (26) found slightly different insulin binding affinities for IR-A and IR-B (2.8 and 1.4 nM, respectively) and lower affinity of IGF-1 for IR-A (120 nM) than we show in Table 1, or Novo Nordisk team reported very high binding affinities of hormones (e.g., 18–23 pM affinity of insulin for IR-A and IR-B) for soluble IRs determined by scintillation proximity assay (27). A concise summary of binding affinities of insulin and both IGFs for IR-A and IR-B determined in different laboratories was published by Westermeier et al. (11) and, in general, our data shown in Table 1 fall well into the range of affinities summarized there.

**Table 1:** Typical values of binding affinities (*K*_d_ values) of insulin, IGF-1, and IGF-2 for human IR-A (in membranes of human IM-9 lymphocytes), human IR-B or human IGF-1R (in membranes of mouse fibroblasts) measured in our laboratory using ^125^I-labeled insulin or ^125^I-labeled IGF-1 as tracers.

Hormone/receptor	IR-A (nM)	IR-B (nM)	IGF-1R (nM)
Human insulin	0.2–0.5^a,b^	0.3–0.7^a^	290^a^
Human IGF-1	24^b^	225^b^	0.2–0.3^a,c^
Human IGF-2	2.9–3.0^b,c^	35^b^	2.3^b^

*^a^Adapted from Ref. (23)*.

*^b^Adapted from Ref. (24)*.

*^c^Adapted from Ref. (25)*.

Binding of insulin and IGFs to IR-A, IR-B, and IGF-1R receptors initiates autophosphorylation of the intracellular tyrosine kinase (TK) domains of the receptors. The molecular mechanism of the signal transduction from extracellular hormone-binding domains to intracellular TKs is still unclear and represents a paradigm of signaling through a TK-like family of receptors (28). Understanding of this mechanism is paramount for progress in human molecular biology and health. The structural characterization of the complete TK (IR-like) receptor was listed as a “structure of desire” for the twenty-first century in *Nature* journal (29). Phosphorylated TKs consequently activate specific cascades of intracellular proteins. IR and IGF-1R utilize common phosphoinositide 3-kinase/Akt and Ras/Erk kinase signaling pathways to mediate a broad spectrum of “metabolic” (mostly insulin) and “mitogenic” (mostly IGFs) responses. It is assumed that the specificity of the action of insulin and IGFs is determined mainly by differential expression of the receptors and responsiveness of tissues, but that IR and IGF-1R may differ in the efficiency with which they activate their major substrates, IRS-1 and IRS-2 and Shc, and that this can influence the effectiveness of “metabolic” or “mitogenic” signaling as well (30).

Another interesting study (31) showed that either insulin, IGF-1 or IGF-2, after binding to IGF-1R, regulated a specific group of transcripts, which was not regulated by another ligand. These data support the hypothesis that the nature of specific hormone-receptor interaction can determine the specific response, even through the same receptor. Moreover, recently, Cai et al. (32) showed that the distinct activities of the closely related IR and IGF1R are mediated by their intracellular juxtamembrane region and substrate binding to this region.

Historically, IR-B is considered as a “metabolic” form of IR, while mainly “mitogenic” effects are attributed to IR-A. However, although some earlier studies indicate that IR-A and IR-B can differ in association and dissociation rates for insulin (33–35) and that *K*_d_ values of insulin for IR-A and IR-B may differ slightly (Table 1), it is unclear whether insulin can activate different cellular proteins through binding to IR-A or IR-B. It is not excluded that mainly differential tissue distribution is the cause of the supposed predominantly metabolic character of IR-B and predominantly mitogenic character of IR-A, because IR-B is expressed mainly in adipose and skeletal muscle tissues with an insulin-dependent glucose uptake by an intermediate of the GLUT-4 transporter and in liver, which lacks GLUT-4 but respond metabolically to insulin in many other ways. A lower affinity of IGFs for IR-B could be important for the safe and exclusive action of insulin in the liver (regulation of enzymes involved in glucose metabolism), which must not be altered by IGF binding to IR-B. It is also possible that the slightly lower affinity of insulin for IR-B, and the predominance of IR-B in liver, may be a form of adaptation to protect the liver from over-stimulation, given that concentration of insulin in hepatic portal blood will be higher than those in the periphery. It is also not clear why the predominant form of the IR in the brain is IR-A and not IR-B. It is possible that IR-A in the brain is the receptor, not only for insulin but also for IGF-2, whose important roles in neural tissues become evident (36). Moreover, the expression of IR-A plays an important role in both insulin and IGF-2 signaling in cancer cells and tumors (37, 38).

The first crystal structures of extracellular IR ectodomains (39, 40) and the structures of the first complexes of IR/IGF-1R fragments with insulin (41, 42) or IGF-1 (43) represent a breakthrough in insulin/IGF structural research. They provided the first information on the interaction of the hormones with the Site 1 of the receptors, which consists of L1 domain and αCT peptide, representing the C-terminus of the receptor α-subunit. Figure 1 shows how insulin interacts with the IR L1, CR domains, and αCT-A peptide (42). It appears evident that the flexible part of the C-terminus of the insulin B-chain (residues B25–B30) must detach from the central core of insulin to avoid a steric clash with the αCT peptide and to ensure the effective binding with the receptor. Consequently, the C-terminal residues 717–719 of the αCT accommodate and partly cover insulin residues B25–B27 (positioned on the L1 domain) in a type of cavity (Figure 1). Insulin residues B28–B30 are invisible in the complex.

**Figure 1:**
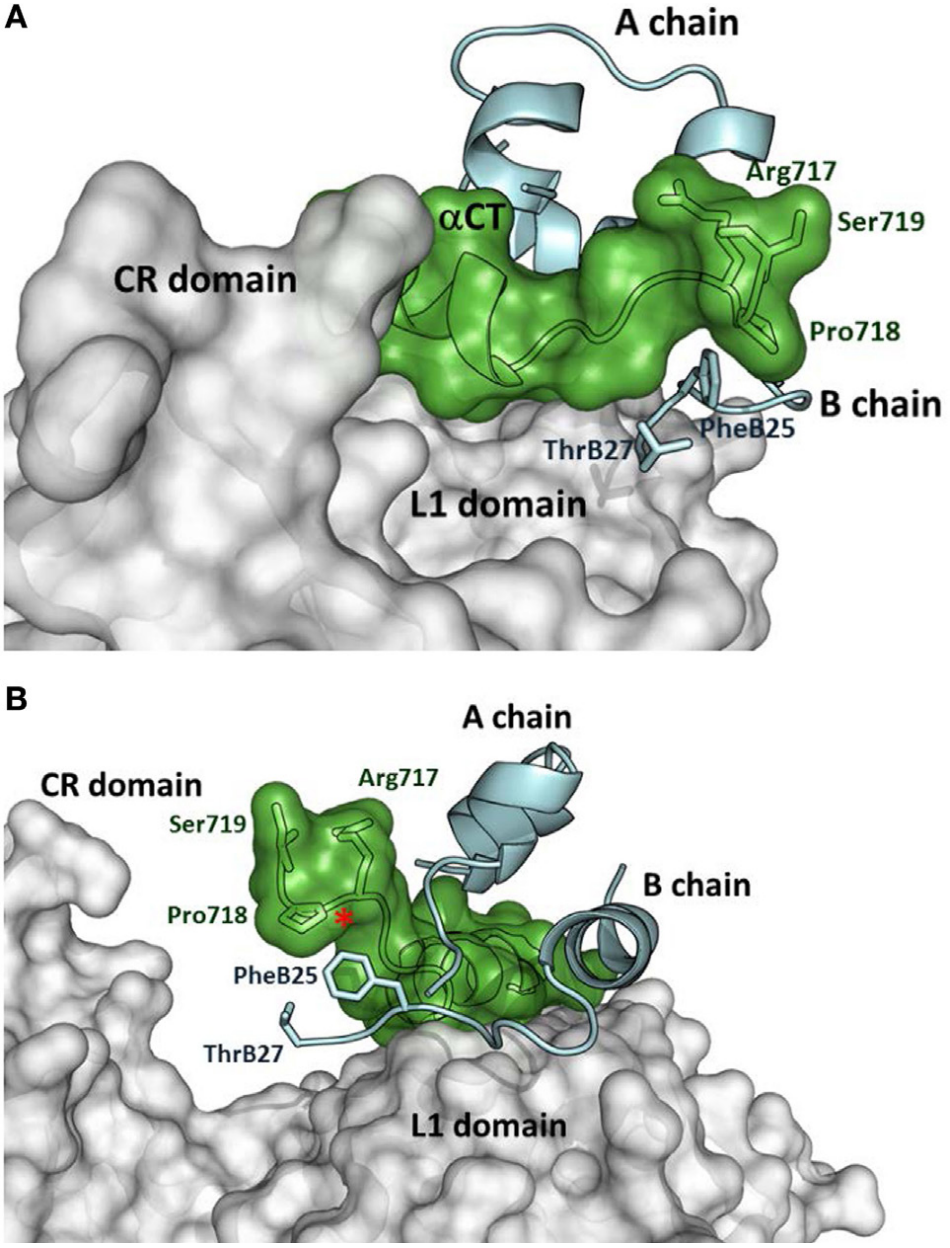
Insulin interaction with L1 and CR domains and αCT-A peptide representing Site 1 fragment of the insulin receptor (IR). **(A)** Detailed view of the C-terminus of the αCT-A (in green) interacting with the receptor L1 domain (in gray) and insulin A- and B- chains (in cyan). The adjacent CR domain of the receptor is also shown (in gray). IR and insulin residues discussed in the text are shown as well. **(B)** The rotated view of the same complex as in **(A)**. The red asterisk shows the insertion site for 12 extra amino acids of αCT-B. The figure was created in PyMol from the PDB ID 4OGA structure (42).

Interestingly, IGF-1 in the complex with IR L1 domain, and the IGF-1R αCT peptide (43) occupy a position, which is superimposable (Figure 2) on the position of human insulin in the previously discussed complex (Figure 1). However, the IGF-1 C domain and the C-terminus of the B domain are not visible in this structure and it is unclear what the receptor-bound position is of these residues (amino acids 28–40, connecting the last visible amino acids Asn26 and Thr41). Closer examination of the complex in Figure 2 reveals the peculiar mutual positioning of the last visible IGF-1 B domain residues (Tyr24–Asn26) and αCT. The B domain residues 24–26 are embedded in a cavity formed by αCT C-terminal segment Val-Pro-Arg-Pro-Ser (IGF-1R numbering 691–706, the last two amino acids are invisible in the complex) and L1 amino acids Asp12, Arg14, and Asn15. However, on the contrary, the last IGF-1 C domain residue Thr41 and the adjacent A domain Gly42 and Ile43 residues “overlie” the αCT peptide (Figure 2).

**Figure 2:**
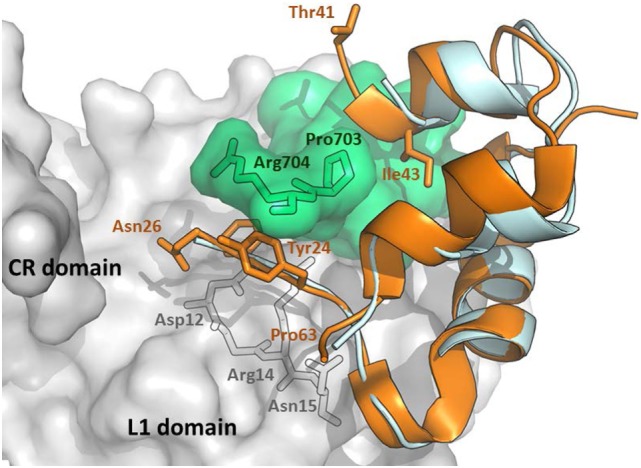
An overlay of receptor-bound structures of IGF-1 and insulin. The hybrid receptor is represented here by the Site 1 fragment composed of L1- and CR-domains of IR and αCT peptide of IGF-1R. The cartoon was created in PyMol from the PDB ID 4XSS complex (43) and overlaid with insulin extracted from the PDB ID 4OGA complex (42). IR CR- and L1 domains are shown in gray, IGF-1R αCT is shown in green, human IGF-1 is in orange and human insulin is in cyan. The residues discussed in the text are labeled in black, orange, or gray.

Menting et al. (43) proposed two different arrangements of the “invisible” IGF-1 residues in their complex. In a model shown in Figure 3, the IGF-1 C and B domains are in a rather extended conformation (dashed orange loops in Figure 3A), partly reminiscent of a crystal structure of free IGF-1 (44) (Figure 3B), and directing the C domain toward the receptor CR domain. This model could be supported by the report indicating the possible interaction of IGF-1 C domain with IGF-1R CR domain (45). The variants where the IGF-1 C domain are not positioned tightly on the surface of the L1 domain could be supported by the absence of the ordered C domain structure in the PDB ID 4XSS model (43). In another model shown in Figure 3A and indicated by black dashed arrows, the IGF-1 B and C domains would be turned back to the A domain of the hormone. However, all these structural possibilities evoke questions about the mutual positioning of the IGF-1 C domain and αCT peptides (especially αCT-B) and about the mechanism and movements by which this complex is formed.

**Figure 3:**
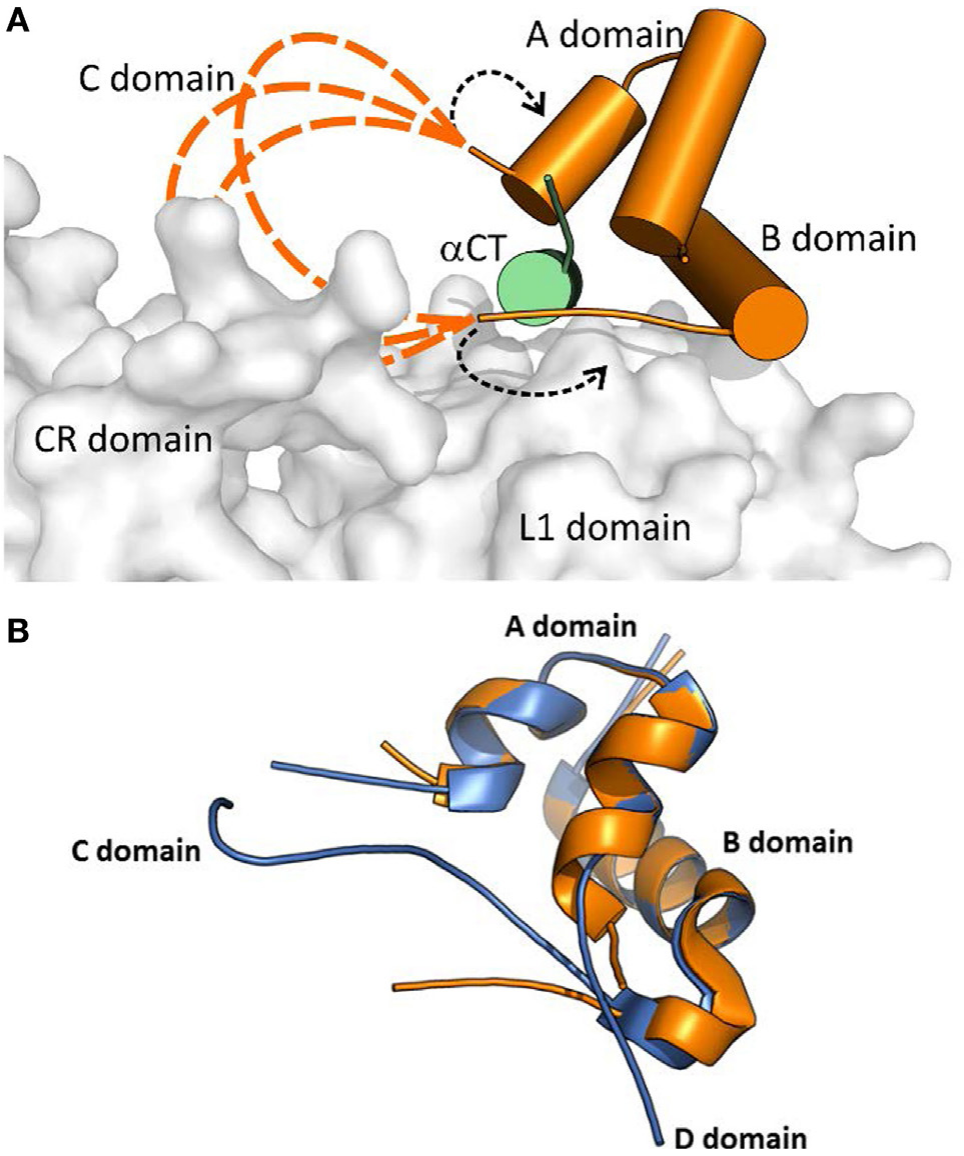
A cartoon showing putative conformations of IGF-1 C domain residues in a hormone:receptor complex. The receptor fragment is the same as shown in Figure 2. **(A)** Receptor-bound IGF-1 is shown in orange. IR L1 and CR domains are shown in gray and IGF-1R αCT in green. The proposed putative positions of IGF-1 C domain residues are shown as dashed orange lines. The alternative positioning of IGF-1 C domain residues is indicated by dashed black arrows. The cartoon was created in PyMol from the PDB ID 4XSS structure (43). **(B)** An overlay of the receptor- bound structure of IGF-1 (in orange, from the PDB ID 4XSS complex) and a crystal structure of human IGF-1 (in blue, from the PDG ID 1GZR structure). The disconnected C domain in the IGF-1 PDB ID 1GZR structure is due to a lack of electron density around positions 36 and 37 (44).

An important aspect of the structures shown in Figures 1 and 2 is that they contain short versions of αCTs, i.e., peptides from IR-A and from IGF-1R. The red asterisk in Figure 1B indicates the position of the insertion of the 12 extra amino acids of IR-B, the only difference between IR-B and IR-A. This 12-amino acid fragment of IR-B is certainly the reason for the lower binding affinity of IGF-1 and IGF-2 toward this receptor isoform. It is indeed rather difficult to imagine that the IGF-1 or IGF-2 C domains could interact with IR-B similarly as shown in Figure 3A, where the C-terminal residues of the short αCT-A go through the loop formed by IGF-1 C domain and B domain. It seems more probable that the longer αCT-B (or its C-terminal part) might be positioned differently on the L1 domain than αCT-A and that it does not go through the IGF C domain loop. A similar hypothesis has already been outlined by Menting et al. (43) and is schematically shown in Figure 4. The speculative cartoons in Figure 4 propose that IR-B αCT could be positioned in a direction toward the CR domain and could interact with the L1 domain or overlie it. This arrangement would allow the positioning of IGF-1 C domain on the αCT-B and would avoid the necessity of putting the long αCT-B peptide through the C domain loop.

**Figure 4:**
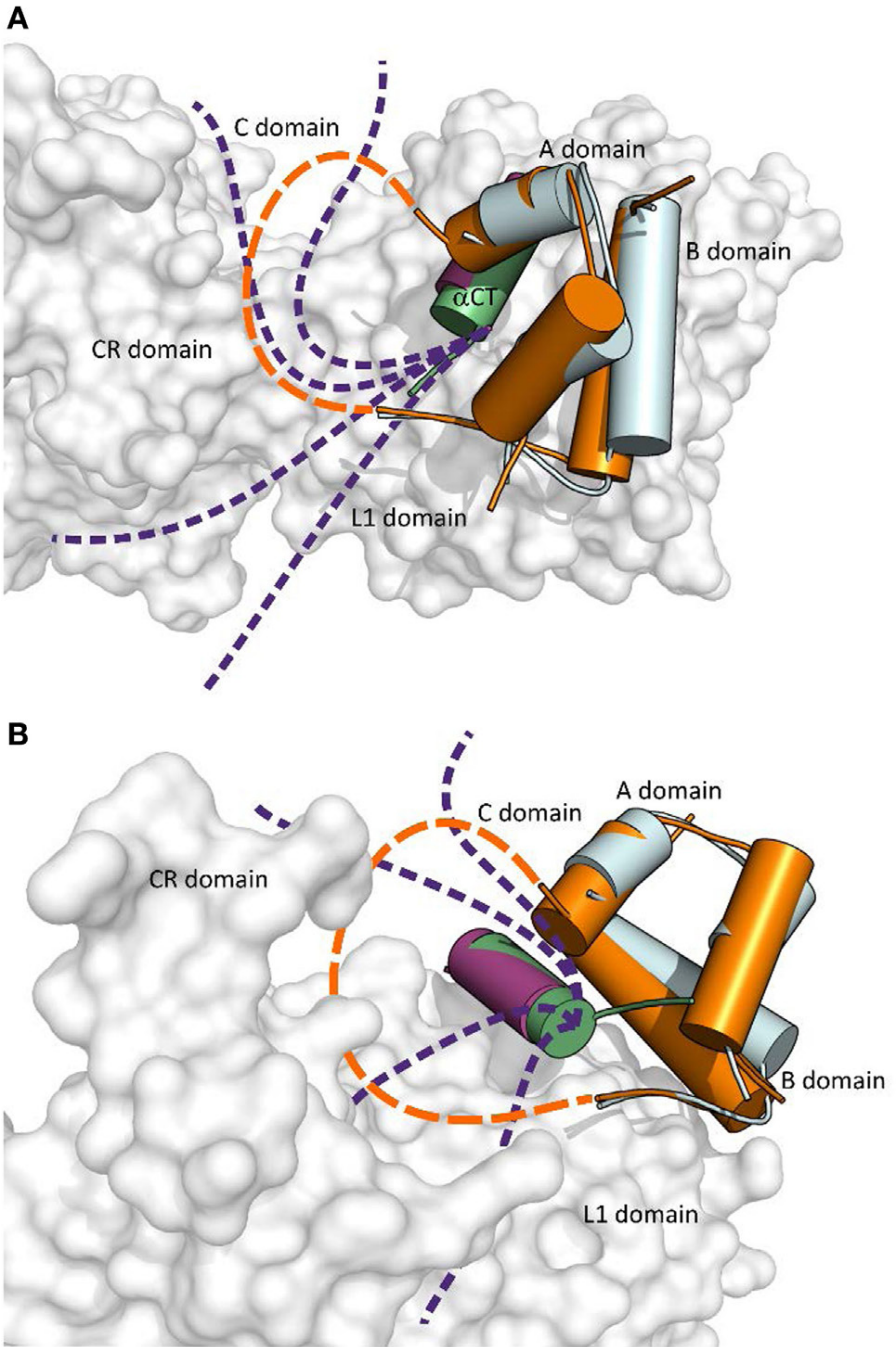
The cartoon showing putative mutual arrangement and positioning of the IGF-1 C domain and C-terminal αCT-B residues on the IR-B receptor. **(A)** An overlay of receptor-bound conformations of human insulin (in cyan, from the PDB ID 4OGA) and human IGF-1 (in orange, from the PDB ID 4XSS). IR L1 and CR domains are in gray, IR αCT-A is in purple and IGF-1 αCT is in green. The dashed orange connecting line indicates a putative position of IGF-1 C domain residues and the purple dashed lines indicate a putative positional range of αCT-B C-terminal amino acid residues, which overlie the L1 domain and are covered by the C domain loop. **(B)** Rotated view of the cartoon shown in **(A)**. Prepared in PyMol.

A specific interaction of insulin analogs with the 12-amino acid αCT-B segment could possibly represent the keystone for the development of IR-B selective insulins. However, advances in these efforts are hampered by the lack of any structural data indicating the position of IR-B in the complexes. Nevertheless, the biological data obtained with insulin and IGF analogs could be useful in the prediction or design of specific interactions of the αCT-B segment. The Novo Nordisk A/S research group presented the first attempts at designing IR-B selective insulin analogs in 2011 (10, 46). They prepared a series of analogs modified at positions A8, B25, and B27. The (HisA8, AsnB25, GluB27-desThrB30)-insulin displayed about 26–30% of binding affinity for IR-B and 8–10% for IR-A (10, 46), which resulted in approximately fourfold enhanced IR-B/IR-A binding selectivity, in comparison with human insulin. Interestingly, it seems that the binding preference of this analog for IR-B was achieved rather by diminishing its binding affinity for IR-A than by enhancing it for IR-B. Detailed examination of the PDB ID 4OGA complex (42) (Figure 1) reveals that insulin Phe at position B25 is in the proximity of αCT-A residues Pro718 and Arg717, while ThrB27 is without any apparent interactions. In the putative complex with IR-B, Pro718 and Arg717 would probably be replaced with Lys718 and Thr719, respectively. It seems that (HisA8, AsnB25, GluB27-desThrB30)-insulin prefers the second possibility. This result indicates that modifications of the C-terminal B25–B27 segment of insulin can lead to an altered receptor-binding specificity.

Interestingly, Novo Nordisk research team recently published an elegant mutagenesis study (27) showing that a single different amino acid at the position 718 of the IR (Lys718 in IR-B versus Pro718 in IR-A) is behind low binding affinities of IGFs for IR-B. This result indicates that relatively subtle structural differences may have important effects on binding affinities and could provide important clues for the design of new more selective ligands.

We recently published the binding affinities of AsnB26-insulin, which has moderately altered binding specificity in favor of IR-B (1.7-fold), while still having native binding affinity (140% for IR-B) (47). This result indicates that changes at B26 can have an impact on the binding specificity as well. Interestingly, the crystal structure of the AsnB26-insulin B24–B28 segment is similar to the receptor-bound conformation of this segment in human insulin (Figure 5) and does not provide a simple clue to the origins of its enhanced IR-B specificity. In another study (23), we prepared a series of insulin analogs specifically crosslinked at different positions of the B24–B29 insulin segment using triazole linkers of different lengths (Figure 5). Interestingly, an analog with a specific linker between positions B26 and B29 had a very high affinity for both isoforms of IR, but with a significant preference for IR-B (217% for IR-A and 570% for IR-B in respect to human insulin; IR-B/IR-A about 2.6-fold). This result indicates that the introduction of artificial chemical motifs, which significantly enlarge the chemical space occupied by this part of insulin, can have an important impact on an analog’s IR-binding properties, i.e., on some hypothetically favorable interaction with αCT-B segment.

**Figure 5:**
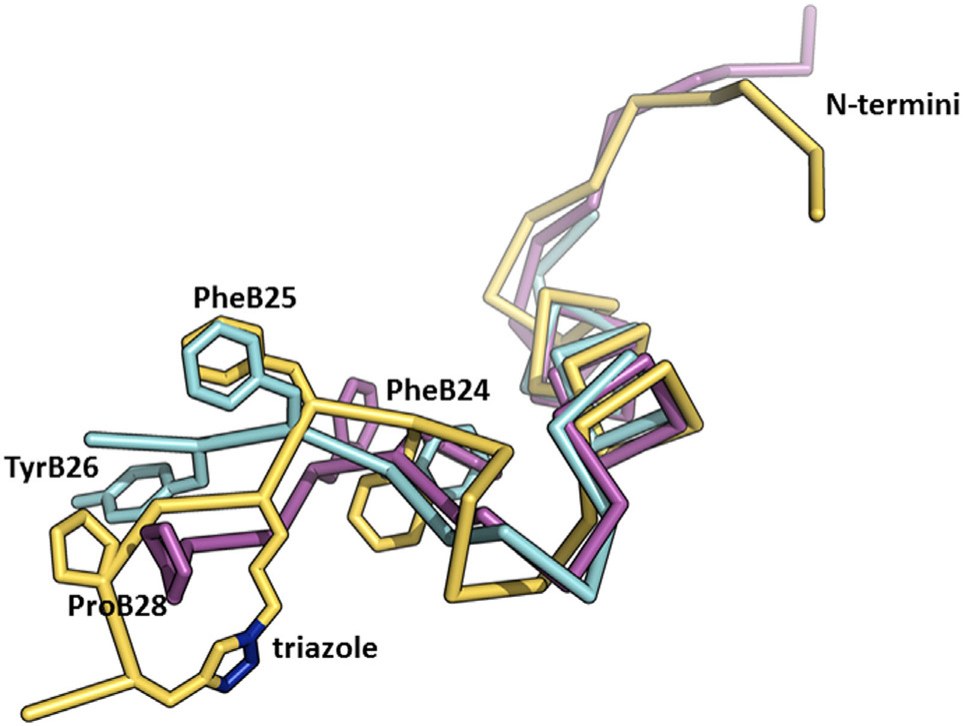
An overlay of the structures of receptor-bound insulin, AsnB26-insulin, and insulin analog with a triazole linker between position B26 and B29. The crystal structure of the receptor-bound insulin (in cyan, from the PDB ID 4OGA) (42) was superimposed on the crystal structure of AsnB26-insulin (in purple, from the PDB ID 4UNG) (47) and on the NMR structure of an insulin analog (in yellow, from the PDB ID 2N2W) (23) with a triazole crosslink (the nitrogen atoms are depicted in blue) between positions B26 and B29. Only the B-chains are shown for better clarity.

Closer inspection of the orientation of the receptor-bound B24-B27 segment of insulin (Figure 1) shows that the B-chain C-terminus of insulin is orientated almost perpendicularly in respect to the orientation of the αCT-A. It can be expected that a similar orientation will be adopted by the B24–B29 segments of the analogs discussed above (23, 47) (Figure 5). If the modifications at the B24–B29 chain of analogs provide some specific and favorable contacts with αCT-B, then this 12-amino acid segment should be oriented on the L1 domain in a similar direction to enable contacts of both peptide chains. This hypothesis would be in agreement with the putative positions of αCT-B proposed in Figure 4.

A flexible D domain of IGF-1 is invisible in the complex with the L1-CR/αCT domains (PDB ID 4XSS). However, it cannot be excluded that the D domain may be involved in some contacts with the C-terminal part of αCT because of their relative proximity (e.g., the distance between Cαs of Arg704 (αCT) and Pro63 (IGF-1), the last visible residues in the complex, is only about 9–10 Å). Recently, we prepared a series of insulin analogs having the C-terminus of the A chain prolonged with D domains of IGF-1 or IGF-2 (24). Interestingly, the analog with the A-chain prolonged for the whole IGF-1 D domain (P^63^LKPAKSA^70^) had similarly diminished binding affinity for both isoforms of IR. This may indicate that the IGF-1 D domain is not in direct contact with the αCT-B extra residues. This would further support the hypothesis proposing that these residues could be positioned in the direction where the C-terminus of the B-chain of insulin is oriented (Figure 4).

We are aware of only one published example of a significantly IR-A selective insulin analog; GlnA18-desThrB30-insulin with A1 and B29 residues connected by VGLSSGQ sequence. This single chain analog, reminiscent of proinsulin or IGFs, has 55% binding affinity for IR-A and 10% binding affinity for IR-B (10). Hence, it seems that the analog’ higher IR-A specificity results from a more negative effect of modifications on IR-B than on IR-A. It is possible that the analog’s VGLSSGQ and adjacent residues could adopt a position similar to the position of the IGF-1 C domain shown in Figures 3 and 4. This mechanism could be behind a lower binding affinity of the analog for αCT-B in analogy with IGF-1.

The Novo Nordisk team (10) used HisA8, AsnB25, GluB27-desThrB30-insulin (26–30% of binding affinity for IR-B and 8–10% for IR-A) and a single-chain insulin analog described above (55% binding affinity for IR-A and 10% binding affinity for IR-B) in rats. These animals have more than 90% IR-B in hepatocytes and in fat cells and more than 90% IR-A in muscle cells. As a consequence, the insulin analog, which has a higher relative affinity for human IR-A, had a higher relative potency (compared with human insulin) for glycogen synthesis in rat muscle (26%) than for glycogen accumulation in rat hepatocytes (5%) and for lipogenesis in rat adipocytes (4%). In contrast, the analog, which has an increased affinity for human IR-B, had higher relative potencies (compared with HI) for inducing glycogen accumulation (75%) and lipogenesis (130%) than for affecting muscle (45%). These results are important, because they indicate that insulin analogs with a relatively moderate IR-isoform preferential binding affinity are able to elicit tissue-selective biological responses, depending on IR-A/IR-B expression.

Finally, the fundamental question is whether it is technically feasible to modify human insulin in a way which will result in it being highly IR-isoform-selective (e.g., with IR-B/IR-A or IR-A/IR-B binding affinity ratio of 100 or more), or whether the selectivity of the currently available analogs has already reached its maximum. Firstly, we do not believe that such hypothetical high binding selectivity (>100) can be reached only by enhancing binding affinity of an analog for IR-B while maintaining near-wild-type affinity for IR-A. Native insulin has an inherently low therapeutic index (the ratio between the doses of the drug that causes an adverse effect relative to the therapeutic dose). Too high receptor affinity of analog could represent a persistent risk for overdosing that can result in life-threatening hypoglycemia or some undesirable growth-promoting effects (1). Moreover, it is technically very difficult to determine properly binding affinities of analogs with more than 7–10-fold enhanced (700–1,000%) binding affinity of human insulin. Therefore, very probably, achieving high binding specificity of an analog will require simultaneous enhancement of its binding affinity for one of the receptors and lowering of its binding affinity for the other. Secondly, we believe that the C-terminus of the insulin B-chain is a promising platform for modifications leading to favorable interactions with CT-B. We have already proven that non-natural modifications of B26 and upstream residues or the prolongation of the chain can result in enhanced affinity and IR-B-specificity. However, these changes are often accompanied by enhanced affinity for IR-A, which lowers the final IR-B/IR-A specificity ratio of the analog. Therefore, some modifications specifically lowering affinity for IR-A would be helpful for higher IR-B specificity. However, identification of such structural determinants is an extremely difficult task. Thirdly, the work of Novo Nordisk (10) has already shown that mimicking the structures of proinsulin and IGFs by connecting insulin chains with peptide sequences can lead to higher IR-A specificity. We believe that this could be a promising method of systematically approaching high IR-A and low IR-B binding. Nevertheless, it appears evident that only a careful design and patient experimental work could lead to some positive responses and results.

## Author Contributions

JJ and LŽ wrote and edited the manuscript.

## Conflict of Interest Statement

The authors declare that the research was conducted in the absence of any commercial or financial relationships that could be construed as a potential conflict of interest.
